# Transmission efficiency of the plague pathogen (*Y. pestis*) by the flea, *Xenopsylla skrjabini*, to mice and great gerbils

**DOI:** 10.1186/s13071-015-0852-z

**Published:** 2015-05-01

**Authors:** Yujiang Zhang, Xiang Dai, Qiguo Wang, Hongjian Chen, Weiwei Meng, Kemei Wu, Tao Luo, Xinhui Wang, Azhati Rehemu, Rong Guo, Xiaotao Yu, Ruifu Yang, Hanli Cao, Yajun Song

**Affiliations:** The Center for Disease Control and Prevention of the Xinjiang Uygur Autonomous Region, Urumqi, 830002 China; Qinghai Institute for Endemic Diseases Prevention and Control, Xining, 811602 China; State Key Laboratory of Pathogen and Biosecurity, Beijing Institute of Microbiology and Epidemiology, Beijing, 100071 China

**Keywords:** *Yersinia pestis*, *Xenopsylla skrjabini*, Flea-borne transmission, Transmission efficiency

## Abstract

**Background:**

Plague, a zoonotic disease caused by *Yersinia pestis*, is characterized by its ability to persist in the plague natural foci. Junggar Basin plague focus was recently identified in China, with *Rhombomys opimus* (great gerbils) and *Xenopsylla skrjabini* as the main reservoir and vector for plague. No transmission efficiency data of *X. skrjabini* for *Y. pestis* is available till now.

**Methods:**

In this study, we estimated the median infectious dose (ID_50_) and the blockage rates of *X. skrjabini* with *Y. pestis*, by using artificial feeders. We then evaluated the flea transmission ability of *Y. pestis* to the mice and great gerbils via artificial bloodmeal feeding. Finally, we investigated the transmission of *Y. pestis* to mice with fleas fed by infected great gerbils.

**Results:**

ID_50_ of *Y. pestis* to *X. skrjabini* was estimated as 2.04 × 10^5^ CFU (95% CI, 1.45 × 10^5^ – 3.18 × 10^5^ CFU), around 40 times higher than that of *X. cheopis*. Although fleas fed by higher bacteremia bloodmeal had higher infection rates for *Y. pestis*, they lived significantly shorter than their counterparts. *X. skrjabini* could get fully blocked as early as day 3 post of infection (7.1%, 3/42 fleas), and the overall blockage rate of *X. cheopis* was estimated as 14.9% (82/550 fleas) during the 14 days of investigation. For the fleas infected by artificial feeders, they seemed to transmit plague more efficiently to great gerbils than mice. Our single flea transmission experiments also revealed that, the transmission capacity of naturally infected fleas (fed by infected great gerbils) was significantly higher than that of artificially infected ones (fed by artificial feeders).

**Conclusion:**

Our results indicated that ID_50_ of *Y. pestis* to *X. skrjabini* was higher than other fleas like *X. cheopis*, and its transmission efficiency to mice might be lower than other flea vectors in the artificial feeding modes. We also found different transmission potentials in the artificially infected fleas and the naturally infected ones. Further studies are needed to figure out the role of *X. skrjabini* in the plague epidemiological cycles in Junggar Basin plague focus.

**Electronic supplementary material:**

The online version of this article (doi:10.1186/s13071-015-0852-z) contains supplementary material, which is available to authorized users.

## Background

Plague, a typical zoonotic disease caused by *Yersinia pestis*, is characterized by its ability to persist in certain natural environments (the plague focus) [1-3]. In the plague natural foci, the pathogen (*Y. pestis*), the vectors (fleas), and the hosts (rodents) interact with each other, which enable the plague to be maintained in the foci for a very long time. *Y. pestis* had been found to infect more than 200 species of wild rodents in the natural foci around the world, and over 80 species of fleas are proven vectors of plague [4].

Fifteen natural plague foci had been identified in China, covering lands more than 1.4 million km^2^ [5,6]. Out of them, focus O, was most recently identified in the Junggar Basin of Xinjiang in 2005 [7]. Extensive field investigation in the Junggar Basin focus had revealed that different kinds of rodents, including *Rhombomys opimus* (great gerbils), *Merionese rythrourus*, *M. tamariscinus*, *Dipus sagitta* etc., and also various kinds of parasitic fleas, including *Xenopsylla skrjabini*, *X. minax*, *X. conformis* and *Echidnophaga oschanini* etc., were involved in the epidemiological cycles of plague in this focus. Notably, most of *Y. pestis* strains (84.4%) isolated in this focus were from the great gerbils and their parasitic fleas, *X. skrjabini* [7,8]. The great gerbils are widely distributed in in sandy or clay desert areas throughout Central Asia (Northwestern China, Mongolia, Russia, Kazakhstan, etc.), and have been confirmed as the major reservoir of some vector-borne zoonoses including plague and leishmaniosis in these areas [9].

The Junggar Basin plague focus is adjacent to Kazakhstan, a known plague focus with the great gerbils and *X. skrjabini* as the main reservoir and vector [10,11]. Therefore, we assumed that the great gerbils and its main parasitic flea *X. skrjabini* should be the reservoir and vector in this plague focus respectively [12]. Although some epidemiological studies had been carried out in Kazakhstan great gerbil foci [13,14], no experimental data is available about the transmission capacity of *X. skrjabini* for *Y. pestis* till now.

To serve as an effective vector for *Y. pestis*, a flea species must be able to take in *Y. pestis* while feeding on an infectious host, to maintain the *Y. pestis* infection until the next bloodmeal, and to transmit acquired *Y. pestis* to a susceptible host during a subsequent bloodmeal [15]. In this paper, we performed preliminary experiments to evaluate the transmission efficiency of *X. skrjabini* for *Y. pestis* to the great gerbils and mice.

## Methods

### Bacterial strain, fleas and animals

*Y. pestis* strain 2505 was isolated from a live great gerbil in the Junggar Basin in 2005 during our routine plague surveillances [16]. It belongs to the biovar Medievalis (positive for glycerol fermentation and negative for nitrate reduction), and its median lethal dose (LD_50_) for mice is less than 10 CFU (colony forming units) [17]. Strain 2505 was cultured, harvested and prepared to certain concentration as previously described [16].

Fleas used in this study (*X. skrjabini*) were captured in Junggar Basin from great gerbils during routine plague surveillances, and they had been reared in the laboratory for more than 10 generations before use [18]. All fleas used in this study were starved for 48 hours right after their eclosions.

BALB/c mice were purchased from Xinjiang experimental animal center. The great gerbils were captured from Junggar Basin and raised in the lab for at least six months. An indirect hemagglutination assay (IHA) and a reverse IHA (RIHA) were employed to screen anti-F1 antibodies and F1 antigens in the gerbils [16], and only those negative for both markers were considered plague-free and suitable for further animal experiments. The animal challenge experiments were performed abiding by the biosafety and ethical regulations issued by the Ministry of Health, China, and approved by the Committee for Animal Welfares of Xinjiang CDC. All experiments involving fleas, mice and great gerbils were carried out in the air-conditioned room at 22°C.

### Median infectious dose of *X. skrjabini* with *Y. pestis*

To determine the median infectious dose (ID_50_) of *X. skrjabini* with *Y. pestis*, six groups of fleas (50 each) were fed for two hours with sterile defibrinated great gerbil blood spiked with the bacteria, which were harvested from Luria-Bertani agar plates grown for 24 hours at 26°C and adjusted to different concentrations (1.0 × 10^8^, 1.0 × 10^9^, 2.0 × 10^9^, 5.0 × 10^9^, 8.0 × 10^9^ and 1.0 × 10^10^ CFU/ml respectively, based on pilot experiment results) within in-house developed feeders. The feeder contained a water-bath to keep the blood at 37°C throughout the feeding. Once the feeding finished, cold water can be pumped into the water bath to cool the feeder, then the fleas will leave the cooled feeder and can be collected by a funnel-like collector into a container (China patent CN 102812927 B).

From each group, 30 to 40 fully fed fleas were harvested right after the 2-hour feeding, and then ground individually and plated on Luria-Bertani agar medium for *Y. pestis* isolation. To exclude potential contamination by unrelated bacteria, plate cultures were confirmed with lysis assays by *Y. pestis* specific bacteria phage Yep-phi (Qinghai Institute for Endemic Diseases Prevention and Control, China) [19]. Fleas with positive culturing results were designated as infected ones, and ID_50_ was calculated as described by Reed and Muench [20], in which the infection rate for certain concentration is set as the cumulative value of groups with concentrations no higher than itself, and ID_50_ is calculated based on the concentrations with cumulative infection rates just higher and lower than 50%. The confidence limit of ID_50_ was estimated by linear regression with SAS 9.3 software (SAS Institute Inc. USA).

### Infection of fleas with different bloodmeal and feeding frequencies

As our previous study showed that *X. skrjabini* feeds at a frequency of 1.6 ± 0.3 per hour [18], we assessed its bacterial acquisition rates resulting from single and multiple blood feedings. To determine the infection rates of *X. skrjabini* with different bacteremia levels and bloodmeal frequencies, two batches of bacteria-spiked great gerbil blood were prepared (1.0 × 10^9^ and 1.0 × 10^10^ CFU/ml respectively). Four groups of fleas (300 each) were fed with the bacterial-spiked blood. The first group was fed with 1.0 × 10^9^ CFU/ml blood for two hours, and the second one with the same bloodmeal three times (two hours each time, with six hours intervals). The third group was fed with 1.0 × 10^10^ CFU/ml blood for two hours, and the last one was fed twice with the same blood (two hours each time with a six hours interval). All groups of fleas were then fed on live uninfected great gerbils for two hours every day, and 20 to 30 fleas from each group were harvested and individually ground for *Y. pestis* isolation. The infection rates for different groups were calculated accordingly.

### Survival and blockage rates of infected fleas

To determine the survival rates of *X. skrjabini* infected with *Y. pestis*, two groups of female fleas (300 each) were fed with bacteria-spiked great gerbil blood (1.0 × 10^9^ and 1.0 × 10^10^ CFU/ml) for two hours within feeders, and then fed on live uninfected great gerbils for two hours every day. Another group of 300 fleas were continuously fed on healthy great gerbils, and set as the control. To estimate the infected rates of fleas, *ca*. 30 fleas from each of the three groups were taken out 24 hours after the first feeding, and tested for the presence of *Y. pestis* (bacteria culturing as previously described). All three groups of fleas were monitored daily for the feeding status, and the unfed fleas were excluded from further analysis. Dead fleas from each group were collected and counted to calculate the survival rates.

To determine the blockage rates of *X. skrjabini*, 600 fleas were fed for two hours with bacteria-spiked great gerbil blood (1.0 × 10^10^ CFU/ml), and then fed on live uninfected great gerbils as previously described. Thirty to fifty fleas were taken out daily for 14 days, and examined microscopically to identify the proventriculus blockages. The blocked fleas had bright red blood in the esophagus but none in the midgut [21,22].

### Bacteria loads dynamics in infected fleas

To determine bacteria loads dynamics in the infected *X. skrjabini*, fleas were fed with bacteria-spiked great gerbil blood (1.0 × 10^10^ CFU/ml) as aforementioned. After the initial infective feeding, the fleas were fed daily with *Y. pestis*-free great gerbil blood, and checked the feeding status daily via microscopic investigation. Unfed fleas were discarded and excluded for further analysis. Five fleas were taken out daily and ground individually for bacterial isolation. Each ground flea was suspended in 2 ml of PBS buffer. The suspension was diluted 1,000 times by PBS, and then 100 μl of the dilution was plated on LB agar media (three plates for each flea). The plates were kept at 26°C for 48 hours and then taken out for *Y. pestis* colonies counting. Suspicious colonies on certain plates were verified by bacteriophage lysis tests.

### Flea transmission of *Y. pestis* to mice via artificial blood feeding

Single-flea transmission of *Y. pestis* by *X. skrjabini* to mice was evaluated via artificial blood feeding systems. Briefly, one group of female fleas (starved for 48 hours) were fed twice (two hours each with six hours of interval), with artificial feeders containing *Y. pestis* spiked defibrinated great gerbil blood (1.0 × 10^10^ CFU/ml). Fleas were checked regularly for the status of feeding, and those without enough red blood in the midguts were regarded as not fully fed and then discarded for further experiments [23]. Thirty fully fed fleas were then reared individually, and fed for two hours daily on the belly of naive BALB/c mice with special feeding capsules (one flea for one mouse). The capsules were made from 5 ml plastic centrifuge tubes with a hole in the lid, and the tube bottoms were cut and covered with gauzes. The fleas were put into the capsules individually, and the capsules were fixed to the naked belly for feeding. Each flea was fed on a new naive mouse every day until the flea died. The mice were reared individually to avoid airborne transmission and direct contact transmission. For the dead mice, livers and spleens were taken out and subjected to *Y. pestis* culturing and phage confirmation tests, while F1 antigens and anti-F1 antibodies were also screened in these gerbils by RIHA and IHA assays [16]. On day 21 post infection (p.i.), the survival mice were sacrificed and subjected to *Y. pestis* culturing, F1 antigen and anti-F1 antibody detection assays as well. Positive results for any of the three tests were considered successful transmissions.

Transmission of *Y. pestis* by multiple fleas was carried out similarly except that 60 fully fed female fleas (12 groups, five in each group) were used to infect the mice. Transmission capacities were evaluated based on the number of infected fleas fed on an individual mouse, and whether or not the transmission was observed for that recipient mouse.

### Flea transmission of *Y. pestis* to great gerbils via artificial blood feeding

In our previous study, we had found out that the average flea index on wild great gerbils is 8.48 (extreme value as 68.1) [7]. Herein we assessed the transmission potential of varied flea numbers on the great gerbils. Fully fed, potentially *Y. pestis* infected fleas were prepared as describe in the previous part. Three groups of naive great gerbils (10 for each group, 30 in total) were subjected to flea infestation, with 10 fleas, 30 fleas and 60 fleas (right after their infectious blood meals) for each great gerbil in the three groups respectively. The gerbils were raised individually in filter-top cages together with the fleas throughout the experiment. F1 antigen (antibody) detection and *Y. pestis* culturing were carried out for the dead gerbils and those sacrificed on day 21 p.i. to screen the transmission events as described in the previous part.

### Flea transmission of *Y. pestis* to mice via great gerbil feeding

Naive great gerbils were infected by subcutaneous injection to the groin, with 1 ml of bacteria-spiked saline (2.0 × 10^10^ CFU/ml) [16]. The great gerbils were reared individually to observe clinical signs for plague (including increased anal temperature, polydipsia, closed eyes, ruffled fur, hunched posture, and lethargy, etc.) [16]. Seventeen gerbils with classical plague symptoms (48 hours p.i.) were selected for flea feeding. For each gerbil, 100 starved fleas were applied to the cage, and fed for 24 hours. Then 20 to 40 fleas from each gerbil were taken out for bacterial isolation and infection rates calculation, while the rest of the fleas were used for mice transmission experiments. *Y. pestis* F1 antigen (antibody) detection and *Y. pestis* culturing were carried out for the dead gerbils or those sacrificed on day 21 p.i. by using similar protocols described in transmission experiments via artificial feeding, and positive results for any of the three above testes were regarded as infected individual.

Transmission of individual great gerbil-fed flea to mice was carried out as aforementioned. In brief, one fully fed flea from each infected great gerbil (12 gerbils in total) was taken out, and applied to one naive mouse every 24 hours. Mice infection was confirmed by *Y. pestis* F1 antigen (antibody) detection or *Y. pestis* culturing as described in the previous parts.

## Results and discussion

### ID_50_ of *X. skrjabini* with *Y. pestis*

Table 1 listed the initial infection rates of *X. skrjabini* fed by artificial bloodmeal with different bacteria concentration. The infection rates are linear correlated with the bacteria concentration in bloodmeal (*R*^*2*^ = 0.89, *p* < 0.01). Based on the data from Table 1, ID_50_ of *Y. pestis* to *X. skrjabini* were estimated as 2.04 × 10^5^ CFU (95% CI, 1.45 × 10^5^ – 3.18 × 10^5^ CFU), given the fact that the median bacteria concentration is 4.08 × 10^9^ CFU/ml and the average bloodmeal for *X. skrjabini* is 0.05 μl [24]. Notably, ID_50_ for *X. skrjabini* is around 40 times higher than ID_50_ previously reported in *X. cheopis* (4.8 × 10^3^ CFU, 95% CI, 2.9 × 10^3^ – 1.4 × 10^4^ CFU) by Lorange et al. [25]. Lorange et al. evaluated flea infection rates after 7 days of *Y. pestis* blood feeding and we tested the instant infection rates of *X. skrjabini* right after the blood feeding. More experiments should be performed to figure out whether the ID50 variations were real or the consequences of different methods.

**Table 1:** **Infection rates of** ***X. skrjabini*** **fed by blood with different bacteremia**

**Bacteremia (CFU/ml)**	Bacteria taken(CFU)*	**Total fleas**	**Infected fleas**	**Infection rate (%)**
1.0 × 10^8^	5.0 × 10^3^	24	4	16.7
1.0 × 10^9^	5.0 × 10^4^	32	13	40.6
2.0 × 10^9^	1.0 × 10^5^	30	13	43.3
5.0 × 10^9^	2.5 × 10^5^	49	25	51.0
8.0 × 10^9^	4.0 × 10^5^	42	27	64.3
1.0 × 10^10^	5.0 × 10^5^	48	36	75.0

*Average bloodmeal volume for *X. skrjabini* was estimated to be 0.05 μl [18].

We were surprised to find out that some fleas were not infected even fed by blood with high bacteria concentration (12 out of 48 fleas not infected with 1.0 × 10^10^ CFU/ml blood). This might be partially explained by the fact *X. skrjabini* will excrete a large proportion of the bloodmeal through anus during the feeding [26]. This characteristic of *X. skrjabini* is quite similar to the adult cat fleas (*Ctenocephalides felis*) [27,28].

In our artificial feeding model, the bacterial concentration ensuring half of *X. skrjabini* to get infected was estimated as 4.08 × 10^9^ CFU/ml (a single bloodmeal), which is higher than the bacteremia level in most of the reported plague animal models (10^5^-10^9^ CFU/ml for rats and 10^8^ CFU/ml for BALB/c mice) [29-31]. Although data about the bacteremia level of plague-infected great gerbils is not available yet, we did report variable bacteria loads in the organs of infected gerbils: 4.1 × 10^4^ to 3.4 × 10^10^ CFU/g in liver and 3.4 × 10^7^ to 1.2 × 10^11^ CFU/g in spleen [16]. The high ID_50_ of *Y. pestis* for *X. skrjabini* implied that a single feed on the infected host might not be enough for this flea to transmit the bacteria efficiently from gerbils with moderate bacteremia level. Our previous study revealed that the blood feeding frequency of *X. skrjabini* is 1.6 ± 0.3 per hour [18], which might be of help to take in more bacteria from the infected hosts.

### Bacteria load dynamics in infected fleas

Table 2 listed the bacteria loads of *X. skrjabini* fed by 1.0 × 10^10^ CFU/ml *Y. pestis* spiked great gerbil blood via artificial feeding. In day 1 p.i., the infection rate of fleas was 80%, with average bacteria loads as 3.16 × 10^5^ CFU per flea, which is close to the amount of bacteria take by the 0.05 μl of bloodmeal (5.0 × 10^5^ CFU) [18]. The average bacteria loads decreased to 4.9 × 10^4^ CFU per flea (10% of the taken bacteria) on day 2 p.i., which implied the rapid digestion of *Y. pestis* by the fleas. On day 3 p.i., the average load rose up to 1.32 × 10^5^ CFU and declined since then. On day 10 p.i., only one of the five fleas was confirmed as *Y. pestis* positive (1.03 × 10^4^ CFU). Our results revealed the clearing and multiplying dynamics of *Y. pestis* in the infected fleas.

**Table 2:** Bacterial loads dynamics in infected fleas*

**Days p.i.**	**Positive rate % (infected/total fleas)**	**Bacterial load (CFU/flea)**
1	80 (4/5)	3.16 ± 5.09 × 10^5^
2	100 (5/5)	4.90 ± 0.65 × 10^4^
3	100 (5/5)	1.32 ± 0.93 × 10^5^
4	80 (4/5)	1.04 ± 1.10 × 10^5^
5	60 (3/5)	6.40 ± 0.67 × 10^4^
6	100 (5/5)	7.30 ± 1.01 × 10^4^
7	80 (4/5)	7.10 ± 0.67 × 10^4^
8	40 (2/5)	8.10 ± 1.08 × 10^4^
9	40 (2/5)	3.20 ± 0.64 × 10^4^
10	20 (1/5)	1.03 ± 0.22 × 10^4^
11	20 (1/5)	4.02 ± 0.80 × 10^3^

*Fleas were fed with great gerbil blood with *Y. pestis* of 1.0 × 10^10^ CFU/ml.

### Effects of bacteremia levels and bloodmeal frequencies on the infection of fleas

**Figure 1:**
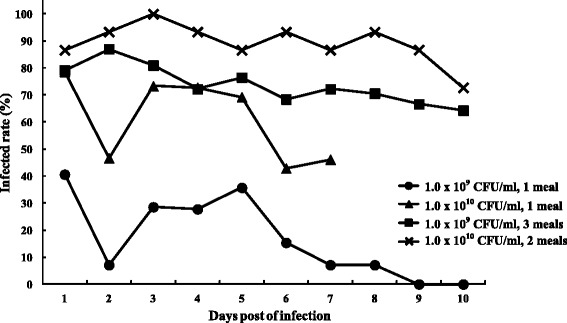
The infection rates of *X. skrjabini* via bloodmeal feeding with different bacteremia levels and feeding frequencies.

As *X. skrjabini* takes bloodmeal at a frequency of 1.6 ± 0.3 per hour [18], we evaluated the effects of bacteremia levels on the infection rates of fleas, and that of feeding frequency as well. We determined the infection rates of *X. skrjabini* to *Y. pestis* by four different feeding schemes: single bloodmeal and triple bloodmeal with 1.0 × 10^9^ CFU/ml, single meal and double meals with 1.0 × 10^10^ CFU/ml. Figure 1 illustrated the flea infection rate curves of different feeding schemes. For both 1.0 × 10^9^ CFU/ml and 1.0 × 10^10^ CFU/ml single-meal groups, the infection rates decrease dramatically in day 2 p.i. (40.6% to 7.1%, and 78.6% to 46.7%), which implied rapid digestion of *Y. pestis* in the fleas immediately after the bloodmeal. On the contrary, infection rates for both multiple bloodmeal groups increase slightly in day 2 p.i. (79.2% to 90.0%, and 86.7% to 93.3%).

In general, single feeding groups had lower infection rates than the multiple feeding ones, and the infection rates kept decreasing during the experiments. The infection rate of the 1.0 × 10^9^ CFU/ml single meal group drop to zero on day 9 p.i. The infection rates of multiple feeding groups remained relatively steady at a high level during the experiments. As *X. skrjabini* will excrete a large proportion of the bloodmeal during the feeding [26], additional infectious bloodmeals might be of help to infect more fleas. Our results demonstrated that not only the initial bacteremia level, but also the feeding frequencies, imposed significant effects on the infection rates of *X. skrjabini* to *Y. pestis.* Multiple feedings would increase the infection rates for the fleas.

### Survival and blockage rates of infected fleas

**Figure 2:**
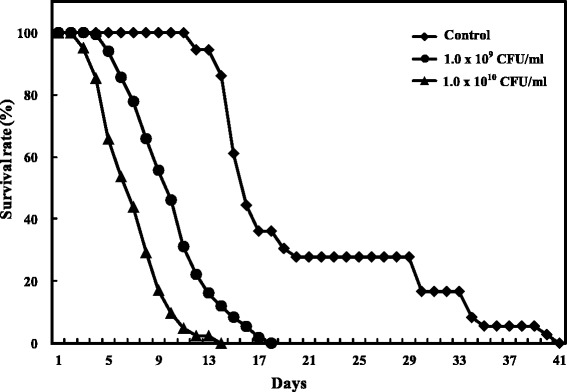
The survival rates of *X. skrjabini* fed by bloodmeals with different bacteremia levels.

We determined the survival rates of female *X. skrjabini* infected by different bacteria-spiked bloodmeals (1.0 × 10^9^ and 1.0 × 10^10^ CFU/ml). As shown in Figure 2, survival rates differed dramatically between the infection groups and the control group. Dead fleas were firstly recorded on day 12 p.i. in the control group, while the survival rates in the 1.0 × 10^9^ and 1.0 × 10^10^ CFU/ml group decreased to 22.4% and 2.4% respectively in the same day. The average life of the control group fleas was 20.67 ± 8.67 days, which was significantly longer than those from 1.0 × 10^9^ CFU/ml group (10.22 ± 3.29 days) and 1.0 × 10^10^ CFU/ml group (7.10 ± 2.57 days) (Independent samples *t* test, *p* < 0.001). Average lives also differed significantly between the two infected groups. Although higher initial bacteremia levels in the blood will increase the infection rates of the fleas (Figure 1), these fleas live much shorter than those fed by lower bacteremia blood (Figure 2).

**Figure 3:**
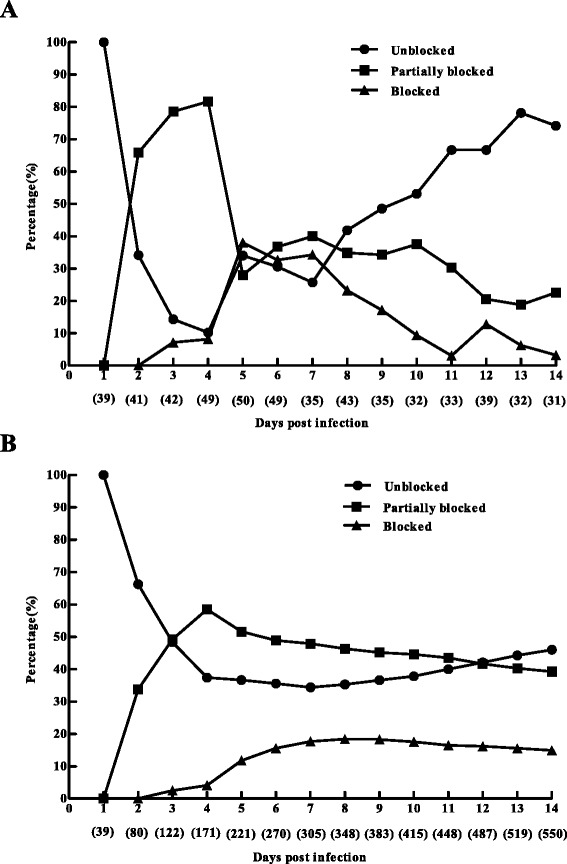
The blockage rates of *X. skrjabini* fed by *Y. pestis* spiked bloodmeal (1.0 × 10^10^ CFU/ml), and the flea numbers were shown in the brackets under the days post infection. **A)**. Blockage status of fleas investigated daily. **B)**. Cumulative percentages of blockages for the investigated fleas (for example, blocked percentage of day 5 p. i. was calculated by the total blocked fleas and total investigated fleas from day 1 to day 5 p. i.).

After fed by 1.0 × 10^10^ CFU/ml bacteria-spiked gerbil blood, the fleas showed three kinds of blockage status: nonblocked, partially blocked and blocked. For the partially blocked fleas, blocks can be seen in the proventriculus while some fresh red blood can be seen in the midgut via light microscopy inspection. The fleas were normally dead after the extensive microscopic observation in this study, therefore we were unable to portrait the temporal blockage dynamics for individual fleas, and we only described the blockage dynamics in the flea population. Figure 3 summarized the blockage rates of fed fleas from day 1 to 14 p.i.

As shown in Figure 3A, a large portion of investigated fleas were partially blocked on day 2 p.i. (65.9%, 27/41 fleas), and then the daily partial blockage rates increased and reached the summit on day 4. p.i. (81.6%, 40/49 fleas). Fully blocked fleas were seen as early as day 3 p.i. (7.1%, 3/42 fleas). The highest daily blockage rate was recorded on day 5. p.i (38.0%, 19/50 fleas), and then the daily rate declined steadily. Our results were similar to the previously reported early blockage formation in *X. skrjabini* (day 2 to day 4) [32], while the mean days to block in *X. cheopis* was reported as 5 to 40 days (normally more than 10 days) [15,33].

Figure 3B illustrated the cumulative percentages of blockages for *X. skrjabini*. The overall blockage rates increased since day 3 p.i, and reached the highest value (18.4%, 64/348 fleas) on day 8 p.i. Although the blockage rate kept relatively steady from day 9 to day 14 p.i., the number of blocked fleas kept increasing as more fleas were included in the calculation. On day 14 p.i., a total of 82 fleas got blocked (14.9%, 82/550 fleas), and this ratio is lower than the observed overall blockage rates of *X. cheopis* (38% to 79%) [15].

Here in this study, we found out that the blocking dynamics of *X. skrjabini* was different from that of *X. cheopis*, such as the early blockages and the lower blocking rate in the former. Our previous work had revealed that the average life of uninfected female adult individual of *X. skrjabini* was 20.50 ± 8.31 days (12 to 40 days) [18], which was shorter than many other kinds of species (more than 50 days for *X. cheopis*). Taking the relatively short life of *X. skrjabini* into account, its early blockage formation might play an important role in the transmission cycles in this plague focus.

In this study, we used dissection microscopes to evaluate the blockage status of *X. skrjabini,* which might be affected by certain subjective factors such as the experiences of the researchers. Recently, a quantitative PCR protocol had been developed to quantify the number of *Y. pestis* bacteria in the fleas, and the authors suggested that bacteria loads higher than 10^6^ per flea might indicate blockages in *X. cheopis* [34,35]. If bacteria loads for *X. skrjabini* blockage are determined, this quantitative PCR method might be applied to verify the blockage data found in this study.

### Plague Transmission of *X. skrjabini* to naive mice via artificial infection

For flea transmission evaluation via artificial infection, we used the high bacteremia feeding scheme (1.0 × 10^10^ CFU/ml, feeding twice via feeders), as it resulted in the highest infection rates for *X. skrjabini* (86.7% to 100% on day 1 to day 3 p.i., Figure 1). Although we did not test the infection status for individual fleas here, we assumed that the vast majority of the fleas were infected by *Y. pestis* based on the results showing in Figure 1.

Among the thirty fully fed fleas for daily individual transmission evaluation, nine fleas died shortly after the artificial bacteria spiked blood feeding, which were then excluded from further study. The other 21 fleas bit a total of 262 mice, seven of which were positive for *Y. pestis* infection (positive rate 2.7%, 7/262). Additional file 1: Table S1 listed the details of the infection status of the 21 fleas and 262 mice. Among the seven infected mice, two were resulted from the biting by the same flea (day 7 and 11 after the artificial bloodmeal of the flea). Five other fleas transmitted *Y. pestis* to one mouse each on day 9, 13, 13, 13 and 18 p.i., respectively. These six fleas bit a total of 82 mice during the experiments, giving an infection rate of 8.5% (7/82) for these mice. In this study, only six out of 21 potentially infected fleas transmitted plague to seven out of 262 bitten mice, which implied poor transmission efficiency of *X. skrjabini* to mice in our artificially infected single-flea mode.

For multiple-flea transmission evaluation of *X. skrjabini* to mice, 12 groups of fleas (five for each group), fed twice by artificial feeder containing *Y. pestis* spiked defibrinated great gerbil (1.0 × 10^10^ CFU/ml), were allowed to fed on 12 naive mice and switched to new mice every day. Additional file 1: Table 3 summarized the results, and Additional file 1: Table S2 listed the temporal dynamics of transmission events. From day 1 to 7 p.i., the fleas effectively bit 73 mice, four of which were infected by *Y. pestis*. The overall infection rate was 5.48% (4/73). Among the 12 groups of potentially infected fleas (a total of 60), only three groups (25%) succeeded in transmitting plague to four mice and one group successfully transmitted plague twice to mice.

**Table 3:** **Plague transmission of** ***X. skrjabini*** to mice via artificial blood feeding*

**Days p.i.**	**Flea used** ^**^	**Mice bit**	**Average flea per mice**	**Infected mice**	**Infection rate (%)**
1	60	12	5	0	0
2	60	12	5	1	8.3
3	60	12	5	1	8.3
4	45	12	3.6	1	8.3
5	28	10	2.8	0	0
6	22	10	2.1	1	10.0
7	8	5	1.6	0	0
Total	/	73	/	4	5.48

*Fleas were infected twice in artificial feeder containing defibrinated great gerbil blood with *Y. pestis* (1.0 x 10^9^ CFU/ml).

**The number of fleas decreased as the flea died during the experiment.

There is no transmission efficiency data available for *X. skrjabini* yet, and the experiment setting in this study (12 groups of fleas repeatedly bit mice everyday) did not allow us to calculate its transmission efficiency of it as what has been done in other fleas [15]. A direct comparison of transmission efficiency of *X. skrjabini* to mice is not feasible with the current dataset. However, an intuitive comparison suggested lower transmission efficiency of *X. skrjabini* to mice than some species of flea*.* For *Oropsylla montana*, almost all groups of potentially infected fleas (96%, 24/25) transmitted plague to mice from day 1 to 4 p.i. [36]. For *X. cheopis* reared at 23°C (similar to the temperature in this study), more than half of the infected flea groups succeeded in transmitting plague to mice (53.7%, 22/41) [37]. A well-designed study, taking various factors (blood sources, feeding schemes, rearing temperatures, timepoints of post infection, etc.) into account [15], will be of great value for estimating the transmission efficiency of *X. skrjabini* accurately.

For the multiple-flea transmission experiments in mice, transmission events were only observed in day 2, 3, 4 and day 6 p.i., when the blockages already occurred in *X. skrjabini* (Figure 3). As shown in Figure 1, less than 20% of fleas got fully blocked and nearly 40% were unblocked on day 6 p.i. Unfortunately, Fleas used in this experiment were kept feeding on mice till their deaths, which prevented us to investigate their blockage status (see the section of *Survival and blockage rates of infected fleas*). Therefore we don’t know whether the transmission events were caused by blocked or unblocked fleas.

Blockage in proventriculus of the infected fleas had been considered the paradigm of *Y. pestis* transmission for a long time [38,39]. However, it was reported that some fleas could also transmit plague efficiently before the blockage formation [36], and the so called early phase transmission (EPT) played vital roles in the transmission cycles of plague for certain fleas [40]. Since EPT refers to the transmission occurring prior to the blockage formation [36] and the transmission events observed here fell into EPT’s time frame, further experiments would be of great help to investigate the role of EPT in the transmission of *Y. pestis* for *X. skrjabini*.

### Plague Transmission of *X. skrjabini* to naive great gerbils via artificial infection

In total 1000 fleas fed with bacteria spiked blood were used to bite three groups of great gerbils (10 gerbils for each group). One group of gerbils were bit with 10 fleas each, while the other two groups with 30 and 60 fleas each. Four great gerbils died unexpectedly soon after the flea biting, within two days due to the malfunction of the cages, which were thought to be deaths unrelated to *Y. pestis* infection (negative for *Y. pestis* isolation and F1-antigen tests) and exclude from further analysis.

Table 4 listed the results for the remaining 26 great gerbils. The 10-flea, 30-flea and 60-flea groups had the infection rates of 44.4%, 40.0% and 71.4% respectively. Our field investigation had revealed that the flea index (flea numbers inhabiting on one host) for the great gerbils is 8.48 (68.1 as the extreme value) [7], therefore data from the 10-flea group might reflect the transmission potentials of *X. skrjabini* to great gerbils more accurately than the two other groups.

**Table 4:** **Plague transmission of** ***X. skrjabini*** to great gerbils via artificial blood feeding*

**Group**	**Gerbil number**	**Flea per gerbil**	**Positive gerbil**	Days to develop disease in gerbils**	**Positive rate (%)**
1	9	10	4	4, 5, 5, 5	44.4
2	10	30	4	6, 7, 7, 7	40.0
3	7	60	5	4, 4, 4, 5, 6	71.4
Total	26	/	13	/	50%

*Fleas were infected twice in artificial feeder containing defibrinated great gerbil blood with *Y. pestis* (1.0 × 10^10^ CFU/ml).

**The time for the great gerbils to develop plague disease after adding the infectious fleas.

As discussed previously, in this artificial feeding model, the fleas were kept feeding on the great gerbils throughout the experiment. We were not able to calculate the transmission efficiency values of *X. skrjabini* for great gerbils, or to determine when the transmission events occurred. The infection rates of great gerbils (40.0-71.4%) were higher than that of mice (5.48%), which implied that *X. skrjabini* might transmit plague more efficiently in great gerbils (its natural host) than in mice. However, different settings were used in these two experiments (flea numbers in each group, feeding schemes, etc.); more studies were needed to estimate their transmission efficiencies for direct comparison.

### Comparison of plague transmission of single *X. skrjabini* via natural infection and artificial infection

We obtained 17 infected great gerbils by subcutaneous injection of *Y. pestis* (2.0 × 10^10^ CFU). To investigate the infection rates of the fleas, 20–40 fleas were harvested from each great gerbil and subjected to *Y. pestis* screening. Fleas from two gerbils were negative from *Y. pestis* infection (21 and 22 fleas respectively), and those from the other 15 gerbils were positive for infection (group positive rate 88.2%, 15/17). A total of 390 fleas from these 15 gerbils were tested, and 325 fleas were confirmed to be infected by *Y. pestis* (flea infection rate 83.3%, 325/390).

We then investigated transmission events of single flea by using 12 naturally infected fleas (infected by great gerbil feeding), which bit a naive mice everyday till their deaths. These 12 fleas bit a total of 156 mice, 22 of which were confirmed to be infected by *Y. pestis* (overall infection rate 14.1%, 22/156, Additional file 1: Table S3). Notably, only 4 of the 12 fleas effectively infected naive mice (bit a total of 50 mice), giving an infection rate as 44.0% (22/50) for the mice bitten by the infective fleas.

Table 5 summarized the single flea transmission results of *Y. pestis* by *X. skrjabini* via artificial infection and natural infection modes. Although the infective flea rates were similar between the naturally infected group and the artificially infected group (6/21, 28.6% vs. 4/12, 33.3%), the infection rates of mice bitten by naturally infected fleas were significantly higher than that of artificially infected ones, both for infective fleas and all fleas (44.00% vs. 8.54% and 14.10% vs. 2.67%, Fisher test, *p* < 0.001). Meanwhile, there is no difference between the average life of the artificial infected fleas and the naturally infected ones. As the bacteremia level can’t be controlled in the natural infection model, we are not sure whether the transmission event differences are caused by the bacteremia issues or the intrinsic characteristics of natural great gerbil plague cycles. More experiments should be performed before we can draw a solid conclusion. Notably, although artificial feeding models is easy for standardization and inter-lab comparisons [15], the differences between mice infection rates of artificial infection model and natural infection model in this study reminded us to take additional thoughts on the results from the artificial feeding models.

**Table 5:** **Comparison of single flea transmission to mice between artificially infected fleas and naturally infected ones**

	**Artificially infected fleas**			**Naturally infected fleas**		
**Group**	**Infective**	**Non-infective**	**Total**	**Infective**	**Non-infective**	**Total**
Number of fleas	6	15	21	4	8	12
Number of mice bit	82	180	262	50	106	156
Number of mice infected	7	0	7	22	0	22
Mice infection rate	8.54%	-	2.67%	44.00%	-	14.10%
Average flea life (days)	14.67 ± 3.88	13.0 ± 3.88	13.5 ± 4.07	15.5 ± 1.83	16.0 ± 2.72	15.83 ± 1.73
Days of infection*	7, 9, 11, 13(3), 18			7(3), 8(4), 9(2), 10(3), 11(2), 12(3), 13(2),15(2), 16(1)		

*Days post of the initial infection of fleas, and the number in the bracket indicating how many mice got infected on that day. 13(3): on day 13 post of flea infection, three mice bit by the fleas get infected (confirmed later depends on the progress of the infection).

In our single-flea transmission experiments for mice, transmission events occurred from day 7 to 16 after the initial flea infection in the naturally infected fleas group, which is quite similar to the pattern of artificially infected fleas (Table 5). Intriguingly, transmission events in mice happened on day 2 to 6 post flea infection in the previously described multiple-fleas artificial infection model (Table 3). As more infected fleas per animal would offer more chances for the bacteria to be transmitted, we can expect the earlier symptoms onsets in the mice with multiple fleas.

We also noticed that, no animal (mice and great gerbil) got infected on day 1 p.i., neither in the artificial infection model nor the natural infection model. Although artificially infected fleas on day 1 p.i. had high bacteria loads (Table 1), they were unable to infect the mice in this study. Our data suggests that the regurgitation or contamination of freshly acquired bacteria into *X. skrjabini* alone might not be enough to cause the infection of a new host. Bacteria multiplying in the fleas might be essential for effective infecting hosts, which probably involves the adaptation of *Y. pestis* in the vector (fleas) and hosts (rodents) [36,38].

## Conclusions

The Junggar Basin plague focus was recently identified in China in 2005, with the great gerbils and *X. skrjabini* as the main reservoir and vector [7,8,18]. Here we performed preliminary experiments aiming to characterize the transmission efficiency of *X. skrjabini* for the plague pathogen, *Y. pestis*, in the great gerbils and mice.

In our artificial feeding model, ID_50_ of *X. skrjabini* with *Y. pestis* was estimated to be 40 times higher than that reported in *X. cheopis* [25]. Although higher bacteremia in the bloodmeal will increase the infection rates *X. skrjabini*, the fleas lived much shorter, which might have less time for transmitting plague among the hosts. We therefore deduced that a single feeding from the infected host might not be enough to make *X. skrjabini* infective, and multiple feedings might be essential for this flea to transmit plague effectively. However, many experiments need to be performed before solid conclusions can be made, such as bacteremia dynamics in the infected great gerbils, bloodmeal consume features of *X. skrjabini*, and well-controlled transmission assessments.

Proventriculus blockage is vital for plague transmission in many species of fleas. Daily blockage tests revealed that *X. skrjabini* got fully blocked as early as day 3 p.i, which was earlier than in other fleas like *X. cheopis*. On the other hand, the overall blockage rate of *X. skrjabini* is lower than that of *X. cheopis*. The differences of blockage dynamics in these two fleas imply that they might have different transmission patterns for plague. There are still some open questions about the blockages features of *X. skrjabini*, such as why it develops blockage so quickly after the infection, how the blockage dynamics affects the transmission efficiency and whether EPT plays roles in the transmission cycles.

We confirmed that *X. skrjabini* is able to transmit plague to mice, and its natural host great gerbils as well, via both artificial infection and natural infection modes. Our results implied that *X. skrjabini* might transmit plague more efficiently to its natural host (great gerbils) than to mice (5.48% vs. 40.0-71.4%) in the multiple-flea feeding schemes. We also noticed that infection rate of mice bitten by naturally infected fleas was significantly higher than that of artificially infected ones in this study, which is worthy of further investigation.

Transmission efficiency tests are very important for us to decipher the role of fleas on transmitting and maintaining the plague in the foci [15,25]. However, these experiments are complicated by the involvements of the bacteria, the flea vector and the hosts, which is quite difficult to interpret [15]. Many parameters, like the lab animals, the blood source, feeding schemes and the temperatures, are known to affect the results [15,37,41]. Notably, we were using fleas right after eclosion throughout this study. Although we found out that there were no significant differences between the volumes of bloodmeals taken by the newly emerged adults and the older ones (data not shown), we are not sure whether the age of *X. skrjabini* will affect plague transmission effects or not. Furthermore, we were not able to quantitatively estimate the transmission efficiencies of *X. skrjabini* to mice and great gerbils. Frankly, we are still far away from figuring out the ecological role of *X. skrjabini* in the Junggar Basin plague focus, and further studies will enable us to get closer to the answer.

## Additional file

Additional file 1: Table S1. Temporal of mice infection of individual flea. **Table S2.** Details of multiple flea transmission tests. **Table S3.** Temporal transmission events of the twelve fleas infected via great gerbil feeding.
