# Supplementary material for: Transmission efficiency of the plague pathogen (Y. pestis) by the flea, Xenopsylla skrjabini, to mice and great gerbils
Source: Parasit Vectors. 2015 May 1;8:256. doi: 10.1186/s13071-015-0852-z (PMC4429828; doi:10.1186/s13071-015-0852-z)
Supplement: Additional file 1: Table S1. — Temporal of mice infection of individual flea. Table S2. Details of multiple flea transmission tests. Table S3. Temporal transmission events of the twelve fleas infected via great gerbil feeding. [file 13071_2015_852_MOESM1_ESM.doc]

Table S1. Temporal of mice infection of individual flea

| Flea index | Flea infected | Mice biting | Infected mice | Infection status of the mice bit daily* | | | | | | | | | | | | | | | | | | | | | |
| --- | --- | --- | --- | --- | --- | --- | --- | --- | --- | --- | --- | --- | --- | --- | --- | --- | --- | --- | --- | --- | --- | --- | --- | --- | --- |
|  |  |  |  | 1 | 2 | 3 | 4 | 5 | 6 | 7 | 8 | 9 | 10 | 11 | 12 | 13 | 14 | 15 | 16 | 17 | 18 | 19 | 20 | 21 | 22 |
| 3 | Yes | 21 | 1 | N | N | N | N | N | N | N | N | N | N | N | N | N | N | N | N | N | Y | N | N | N | D |
| 4 | Yes | 13 | 1 | N | N | N | N | N | N | N | N | N | N | N | N | Y | D |  |  |  |  |  |  |  |  |
| 5 | Yes | 12 |  | N | N | N | N | N | N | N | N | N | N | N | N | D |  |  |  |  |  |  |  |  |  |
| 6 | Yes | 15 |  | N | N | N | N | N | N | N | N | N | N | N | N | N | N | N | D |  |  |  |  |  |  |
| 7 | Yes | 10 |  | N | N | N | N | N | N | N | N | N | N | D |  |  |  |  |  |  |  |  |  |  |  |
| 8 | Yes | 13 |  | N | N | N | N | N | N | N | N | N | N | N | N | N | D |  |  |  |  |  |  |  |  |
| 10 | Yes | 16 |  | N | N | N | N | N | N | N | N | N | N | N | N | N | N | N | N | D |  |  |  |  |  |
| 11 | Yes | 9 |  | N | N | N | N | N | N | N | N | N | D |  |  |  |  |  |  |  |  |  |  |  |  |
| 13 | Yes | 10 |  | N | N | N | N | N | N | N | N | N | N | D |  |  |  |  |  |  |  |  |  |  |  |
| 14 | Yes | 8 |  | N | N | N | N | N | N | N | N | D |  |  |  |  |  |  |  |  |  |  |  |  |  |
| 15 | Yes | 13 | 1 | N | N | N | N | N | N | N | N | N | N | N | N | Y | D |  |  |  |  |  |  |  |  |
| 17 | Yes | 10 | 1 | N | N | N | N | N | N | N | N | Y | N | D |  |  |  |  |  |  |  |  |  |  |  |
| 18 | Yes | 14 | 1 | N | N | N | N | N | N | N | N | N | N | N | N | Y | N | D |  |  |  |  |  |  |  |
| 19 | Yes | 8 |  | N | N | N | N | N | N | N | N | D |  |  |  |  |  |  |  |  |  |  |  |  |  |
| 21 | Yes | 10 |  | N | N | N | N | N | N | N | N | N | N | D |  |  |  |  |  |  |  |  |  |  |  |
| 23 | No | 20 |  | N | N | N | N | N | N | N | N | N | N | N | N | N | N | N | N | N | N | N | N | D |  |
| 24 | Yes | 11 |  | N | N | N | N | N | N | N | N | N | N | N | D |  |  |  |  |  |  |  |  |  |  |
| 25 | Yes | 6 |  | N | N | N | N | N | N | D |  |  |  |  |  |  |  |  |  |  |  |  |  |  |  |
| 26 | Yes | 11 | 2 | N | N | N | N | N | N | Y | N | N | N | Y | D |  |  |  |  |  |  |  |  |  |  |
| 27 | No | 20 |  | N | N | N | N | N | N | N | N | N | N | N | N | N | N | N | N | N | N | N | N | D |  |
| 28 | No | 12 |  | N | N | N | N | N | N | N | N | N | N | N | N | D |  |  |  |  |  |  |  |  |  |

*: N, mice not infected; Y, Mice get infected; D, Flea died.

Table S2. Details of multiple flea transmission tests.

| Days p.i. | Mice bit | Mice infected | Flea fed on individual mice* | | | | | | | | | | | |
| --- | --- | --- | --- | --- | --- | --- | --- | --- | --- | --- | --- | --- | --- | --- |
|  |  |  | 1 | 2 | 3 | 4 | 5 | 6 | 7 | 8 | 9 | 10 | 11 | 12 |
| 1 | 12 | 0 | 5 | 5 | 5 | 5 | 5 | 5 | 5 | 5 | 5 | 5 | 5 | 5 |
| 2 | 12 | 1 | 5 | 5 | 5 | 5 | 5 | 5 | 5 | 5 | 5 | 5 | 5 | 5 |
| 3 | 12 | 1 | 5 | 5 | 5 | 5 | 5 | 5 | 5 | 5 | 5 | 5 | 5 | 5 |
| 4 | 12 | 1 | 5 | 3 | 4 | 5 | 3 | 2 | 4 | 4 | 2 | 3 | 5 | 5 |
| 5 | 10 | 0 | 3 | 2 | 4 | 2 | 3 | 3 | 2 | 4 | 3 | 2 |  |  |
| 6 | 10 | 1 | 3 | 2 | 3 | 2 | 2 | 2 | 2 | 2 | 2 | 2 |  |  |
| 7 | 5 | 0 | 2 | 1 | 2 | 2 | 1 |  |  |  |  |  |  |  |

*: Shaded mice were confirmed infected.

Table S3. Temporal transmission events of the twelve fleas infected via great gerbil feeding

| Flea Index | Mice bit | Mice infected | Mice infection status daily bit by the infected fleas* | | | | | | | | | | | | | | | | | | | |  |
| --- | --- | --- | --- | --- | --- | --- | --- | --- | --- | --- | --- | --- | --- | --- | --- | --- | --- | --- | --- | --- | --- | --- | --- |
|  |  |  | 1 | 2 | 3 | 4 | 5 | 6 | 7 | 8 | 9 | 10 | 11 | 12 | 13 | 14 | 15 | 16 | 17 | 18 | 19 | 20 | 21 |
| 1 | 14 | 6 | N | N | NF | N | N | N | Y | Y | NF | Y | N | Y | Y | N | N | Y | D |  |  |  |  |
| 2 | 15 | 0 | NF | N | N | N | N | N | N | N | N | N | N | NF | N | N | NF | N | N | N | D |  |  |
| 3 | 13 | 0 | N | N | N | N | N | N | NF | N | N | N | N | NF | N | NF | N | N | D |  |  |  |  |
| 4 | 12 | 5 | N | N | N | N | NF | N | Y | Y | Y | Y | NF | N | Y | NF | N | D |  |  |  |  |  |
| 5 | 14 | 0 | N | N | N | N | N | N | N | N | NF | N | N | N | N | N | N | D |  |  |  |  |  |
| 6 | 16 | 0 | N | N | NF | N | N | N | N | NF | N | N | NF | N | N | N | NF | N | N | N | N | N | D |
| 7 | 12 | 0 | NF | N | N | N | N | NF | N | N | N | NF | N | N | N | N | N | D |  |  |  |  |  |
| 8 | 12 | 5 | N | N | NF | N | N | N | N | Y | Y | NF | Y | Y | N | NF | Y | D |  |  |  |  |  |
| 9 | 14 | 0 | N | N | NF | N | N | N | N | N | N | N | N | NF | N | N | N | N | D |  |  |  |  |
| 10 | 12 | 0 | N | N | N | NF | N | N | N | NF | N | N | N | N | NF | N | N | D |  |  |  |  |  |
| 11 | 10 | 0 | N | N | NF | NF | N | N | N | N | NF | N | N | N | N | D |  |  |  |  |  |  |  |
| 12 | 12 | 6 | NF | N | N | N | NF | N | Y | Y | NF | Y | Y | Y | NF | N | Y | N | D |  |  |  |  |

*: Y: Mice infected; N: Mice not infected; NF: Fleas not fed on the mice; D: Fleas died.
